# Assessing the Value of Unsupervised Clustering in Predicting Persistent High Health Care Utilizers: Retrospective Analysis of Insurance Claims Data

**DOI:** 10.2196/31442

**Published:** 2021-11-25

**Authors:** Raghav Ramachandran, Michael J McShea, Stephanie N Howson, Howard S Burkom, Hsien-Yen Chang, Jonathan P Weiner, Hadi Kharrazi

**Affiliations:** 1 Applied Physics Laboratory Johns Hopkins University Baltimore, MD United States; 2 Center for Population Health Information Technology Department of Health Policy and Management Johns Hopkins School of Public Health Baltimore, MD United States

**Keywords:** persistent high users, persistent high utilizers, latent class analysis, comorbidity patterns, utilization prediction, unsupervised clustering, population health analytics, health care, prediction models, health care services, health care costs

## Abstract

**Background:**

A high proportion of health care services are persistently utilized by a small subpopulation of patients. To improve clinical outcomes while reducing costs and utilization, population health management programs often provide targeted interventions to patients who may become persistent high users/utilizers (PHUs). Enhanced prediction and management of PHUs can improve health care system efficiencies and improve the overall quality of patient care.

**Objective:**

The aim of this study was to detect key classes of diseases and medications among the study population and to assess the predictive value of these classes in identifying PHUs.

**Methods:**

This study was a retrospective analysis of insurance claims data of patients from the Johns Hopkins Health Care system. We defined a PHU as a patient incurring health care costs in the top 20% of all patients’ costs for 4 consecutive 6-month periods. We used 2013 claims data to predict PHU status in 2014-2015. We applied latent class analysis (LCA), an unsupervised clustering approach, to identify patient subgroups with similar diagnostic and medication patterns to differentiate variations in health care utilization across PHUs. Logistic regression models were then built to predict PHUs in the full population and in select subpopulations. Predictors included LCA membership probabilities, demographic covariates, and health utilization covariates. Predictive powers of the regression models were assessed and compared using standard metrics.

**Results:**

We identified 164,221 patients with continuous enrollment between 2013 and 2015. The mean study population age was 19.7 years, 55.9% were women, 3.3% had ≥1 hospitalization, and 19.1% had 10+ outpatient visits in 2013. A total of 8359 (5.09%) patients were identified as PHUs in both 2014 and 2015. The LCA performed optimally when assigning patients to four probability disease/medication classes. Given the feedback provided by clinical experts, we further divided the population into four diagnostic groups for sensitivity analysis: acute upper respiratory infection (URI) (n=53,232; 4.6% PHUs), mental health (n=34,456; 12.8% PHUs), otitis media (n=24,992; 4.5% PHUs), and musculoskeletal (n=24,799; 15.5% PHUs). For the regression models predicting PHUs in the full population, the F1-score classification metric was lower using a parsimonious model that included LCA categories (F1=38.62%) compared to that of a complex risk stratification model with a full set of predictors (F1=48.20%). However, the LCA-enabled simple models were comparable to the complex model when predicting PHUs in the mental health and musculoskeletal subpopulations (F1-scores of 48.69% and 48.15%, respectively). F1-scores were lower than that of the complex model when the LCA-enabled models were limited to the otitis media and acute URI subpopulations (45.77% and 43.05%, respectively).

**Conclusions:**

Our study illustrates the value of LCA in identifying subgroups of patients with similar patterns of diagnoses and medications. Our results show that LCA-derived classes can simplify predictive models of PHUs without compromising predictive accuracy. Future studies should investigate the value of LCA-derived classes for predicting PHUs in other health care settings.

## Introduction

A small segment of the patient population utilizes a high volume of health care services [1,2]. Population health management programs often aim to identify high-utilizing subpopulations and provide them with appropriate preventative interventions to reduce undesired health outcomes while lowering utilization [2,3]. Reducing unnecessary health care utilization such as avoidable inpatient admissions enables more effective use of health care resources across the patient population, hence improving the overall health of the managed population [2-4].

Population health programs are often managed by insurers and health care providers [2,5]. Traditionally, health care payers use insurance claims to identify members/enrollees with high rates of utilization. Health care providers are increasingly using electronic health records (EHRs) to identify high-utilizing patients [6,7]. Payers and providers routinely apply established risk stratification techniques against their data to predict the members/patients who will become a high utilizer in the short term (eg, 30 days to 12 months) [8-11]. However, predicting who will continuously remain a high utilizer in the long term (eg, 24 months or more) has proven to be a challenging task for population health risk stratification [12].

Persistent high users/utilizers (PHUs) are patients who have a high utilization rate over an extended period (eg, a patient whose annual costs are in the top 20% of all patients’ costs over 4 consecutive 6-month periods) [1,13]. Recent studies have taken several approaches to characterizing PHUs, including the frequency and type of utilization, total costs, and number of chronic conditions [1,8-13]. Despite the variety of terminologies used for PHUs (eg, high-cost high-need, super-utilizers), population health analysts have typically faced barriers in extracting the common probability classes of diagnoses and medications for PHUs to improve the management of health care resources in specific subpopulations [13,14].

PHUs constitute a small percentage of the patient population [1]. PHUs of a health system may present a different mix of comorbidities and medications compared with those of PHUs in other health systems [8-14]. The variability of the underlying probabilities of PHUs’ diseases and medications across different settings complicates the use of traditional approaches for identifying PHUs from groupings of diagnostic codes. Considering this diversity of conditions, the manual grouping of diagnostic and medication codes by clinical experts will not only be burdensome to compile for a given health system but also impractical to use elsewhere [1-3]. Automated clustering/grouping techniques can be a valuable alternative to characterizing PHUs for a specific health system patient subpopulation [15-19]. Automated groupings of health care utilization patterns can also enhance the prediction of PHUs through traditional analytical methods such as logistic regression [15].

To address the difficulties of identifying common patterns of comorbidities among PHUs, in this study, we implemented an unsupervised clustering methodology, latent class analysis (LCA) [20], to semiautomatically classify PHU patients by a limited number of probability classes of characteristic comorbidities and medications. We then used the LCA classes along with a few demographic and health system factors to predict PHU status for each member of the total study population and a selected set of patient subpopulations. We finally compared our LCA-enabled predictive model with a sophisticated (but more complex) risk stratification model that uses several demographic, clinical, and medication factors to predict PHU status.

## Methods

### Overall Aims and Definitions

The overall goal of our study was to identify subpopulations of PHUs where changes in care delivery could reduce the risk of high utilization. Our analysis aimed to automate the extraction of common probabilistic patterns of comorbidities and medications for PHUs, and then use such information to improve the prediction of PHUs among the study population as well as specific diagnostic subpopulations.

We defined a PHU as an individual whose medical charges remained in the top 20% of the highest health care costs for 4 consecutive 6-month periods (ie, total of 2 years after the base period) [1]. Health care costs were defined as the sum of hospital inpatient, outpatient department, emergency department (ED), and professional and pharmacy costs covered by the insurer and the patient’s out-of-pocket costs [1,6].

### Data Source and Preparation

We performed a retrospective analysis of the Johns Hopkins Health Care (JHHC) insurance claims data captured between 2013 and 2015. JHHC provides health insurance to a variety of enrollees, including Medicaid and employer-based members. JHHC enrollees can also seek care outside of the Johns Hopkins health system. We applied the Johns Hopkins Adjusted Clinical Groups (ACG) software to the claims data to generate additional health care utilization variables consistent with previous PHU analyses [1,21]. We categorized the diagnostic codes into higher-level diagnosis groupings defined by the ACG methodology as expanded diagnostic clusters (EDCs), and grouped the medication data into ACG prescription-defined morbidity groups (RxMGs) [21]. EDCs and RxMGs, which are extensively validated and routinely used for risk stratification [1,6], were used in our analysis as the base diagnosis and medication categories, respectively.

### Study Population

**Figure 1:**
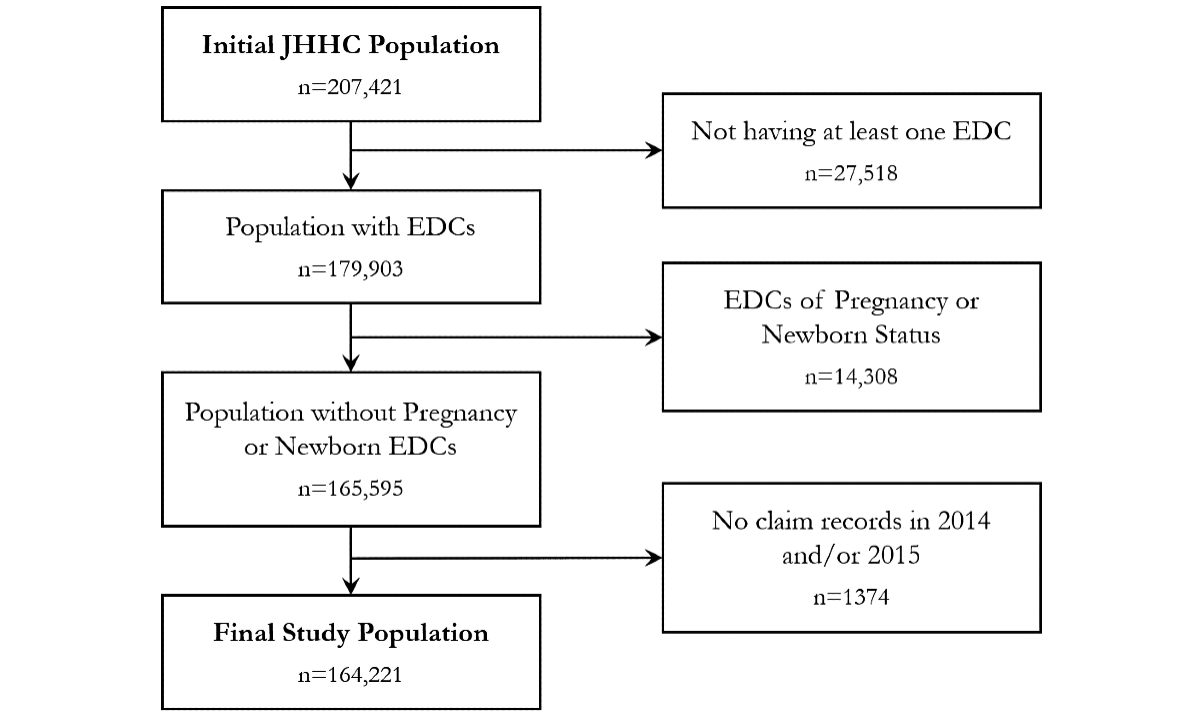
Selection process of the study population. JHHC: Johns Hopkins Health Care; EDC: expanded diagnostic cluster.

Our initial sample population included 207,421 patients with at least one JHHC claims record in 2013 and at least 2 years of continuous JHHC enrollment between 2013 and 2015 (Figure 1). Following the CONSORT (Consolidated Standards of Reporting Trials) statement [22], we first excluded 27,518 patients with missing EDC diagnosis codes since EDCs were used to identify clusters of patients within the population. Next, we excluded 14,308 patients with pregnancy or newborn EDC codes since high costs typical of pregnancy complications differ from those that distinguish PHUs. Finally, we excluded an additional 1374 patients without JHHC claims in 2014-2015 since data in 2013 were used to predict PHUs in 2014 and 2015. The final study population included 164,221 patients (Figure 1).

To explore the sensitivity of our approach, we further divided the study population into four distinct diagnostic-driven subpopulations. These subpopulations were chosen based on the frequency of the underlying EDC data and were validated by two clinicians. The clinicians reviewed the combination of EDCs and asserted their practical use in clinical settings. These subpopulations were identified as: (1) otitis media (n=24,992 patients), (2) mental health (n=34,456), (3) musculoskeletal signs and symptoms (n=24,799), and (4) acute upper respiratory infection (URI; n=53,232).

### Predictors and Outcome

The full study population and each subpopulation contained several predictor variables and the outcome variable. Predictors (ie, independent variables) included demographics, EDCs, Rx-MGs, and other health utilization variables (eg, hospitalization, care coordination) generated by the ACG system. Many of these predictors, including all EDCs and Rx-MGs, are categorical variables [21].

The outcome of interest, a binary variable, was whether or not a patient became a PHU after the base year (ie, being in the top 20% of the highest health care costs over 4 consecutive 6-month periods from 2014 to 2015). The outcome variable was calculated separately in the full population and in each of the diagnostic subpopulations (eg, a patient might be considered a PHU in a subpopulation but not in the full population).

### Statistical Approach

#### Unsupervised Clustering to Identify Diagnoses Clusters

LCA was performed on the full study population and on each subpopulation separately to identify “phenotypes” (ie, classes) of disease subtypes [20]. LCA is an unsupervised data-driven clustering technique that identifies unobserved subtypes (latent classes) within a population based on probability theory. A key assumption in LCA is that conditional independence (ie, latent class membership) explains all of the shared variance across variables [20].

The main parameters generated by LCA are the probabilities of latent class membership for each individual (ie, each patient in the mental health subpopulation; n=34,456) and the class-specific probabilities of observing each binary variable (eg, tobacco use EDC among mental health patients). These probabilities distinguish LCA from binning techniques in which each individual (eg, patient) is merely assigned a probability of belonging to an unobserved/latent class (eg, representing a specific pattern of comorbidities) based on a well-established statistical theory [20].

LCA creates latent classes that optimize minimizing the variance across individuals within each class while maximizing the variance between individuals in different classes. Moreover, LCA is a person-centered approach, does not make distributional assumptions, and works well with categorical data, making it particularly applicable to subtype identification of patients using diagnostic data such as EDCs [20].

LCA models with a varied number of latent classes (2 to 6 classes) were constructed using EDC, Rx-MG, and selected patient-level resource utilization variables. For both the full population and the select subpopulations, 4-class models were chosen because they provided the right balance between optimal model fit and interpretability of the classes. Although models with more classes (eg, 5- and 6-class models) might fit the data slightly better, the interpretation of the classes becomes less clear, and often classes may differ only across a few variables. In other words, the gain in fit is not sufficient to overcome the decline in interpretability that comes from adding too many classes to the model. Additionally, LCA models with more than 6 classes did not improve the standard fit metrics, explained a very small proportion of patients, and had limited mathematical convergence, and were therefore not considered in this study.

LCA fit was measured using G^2^, Akaike information criterion (AIC), and Bayesian information criterion (BIC) metrics; lower values of G^2^, AIC, and BIC imply a better fit [23,24]. Similar to standard regression techniques, LCA uses maximum-likelihood estimation to determine its model parameters. The goal of maximum-likelihood estimation is to maximize the probability (likelihood, *L*) that the process described by the model produced the observed data: G^2^=–2×log(*L*), AIC=–2×log(*L*)+2×*k*, and BIC=–2×log(*L*)+*k*×log(N), where *k* is the number of estimated model parameters and N is the sample size. Since *L* is maximized to achieve the best fit to the data, –2×log(*L*) is also minimized, and thus lower G^2^, AIC, and BIC values indicate a better model fit. For a large sample where log(N)>2, AIC tends to favor more complex models (ie, more model parameters) over BIC [23,24].

LCA does not bin each individual into a class but rather calculates the probability that an individual’s characteristics most closely match those of the other individuals in each class. Classes are constructed to maximize similarity of individuals’ characteristics within a class and dissimilarity of individuals across classes. For example, in this study, the LCA methodology generated four different class probabilities for each patient representing the similarity of the patient’s comorbidities (ie, mix of EDCs and RxMGs) to comorbidities of patients in each LCA-derived class of the entire study population.

#### Logistic Regression Modeling to Predict PHUs

Once the classes were constructed via LCA and health utilization characteristics of the classes were graphically compared, we trained logistic regression models to predict PHUs in both the full population and in each subpopulation using the following variables: (1-3) latent class membership probabilities for 3 of the 4 classes (the class with the lowest chronic EDC/RxMG probabilities was chosen to be the reference class); (4) gender (male; reference=female); (5-9) race (Black, Asian, Hispanic, other, missing; reference=White); (10) medical and pharmacy coverage in 2013; (11) Medicaid eligibility; (12) number of acute care inpatient days; (13) number of acute care inpatient stays; (14) presence of frailty conditions; and (15-16) likely or possibly experiencing care coordination issues (yes/no). Variables 12 to 16 were generated by the ACG system [21] using the JHHC medical claims data.

We also used the ACG system’s internal risk stratification functions (ie, embedded models) to predict PHU status in the full population [21]. The ACG system implements a complex model that uses over 300 variables (eg, demographics, all EDCs, all RxMGs, and dozens of health system variables) to predict health care utilization such as inpatient admissions, ED visits, and overall medical or pharmacy costs. Predictive performance of all regression models was assessed and compared using sensitivity, predictive positive value (PPV), and the F1-score.

All analyses, including the descriptive analysis of the full population and all subpopulations, were performed in R (v3.5.1). We used R’s basic packages for the LCA clustering [25] and logistic regression predictions.

## Results

### Descriptive Analyses

Descriptive statistics for the full population are summarized in Table 1. Overall, approximately 5% of the full population were identified as PHUs. The average age of PHUs was more than twice that of the non-PHU population. The percentage of males was smaller among PHUs than among non-PHUs. As expected, a larger percentage of PHUs had one or more inpatient or outpatient visits compared to non-PHUs (18.7% vs 2.5% for inpatient visits and 99.7% vs 97.3% for outpatient visits, respectively). Similar descriptive statistics were generated for each of the four diagnostic subpopulations (see Multimedia Appendix 1-4).

**Table 1:** Characteristics of the study populations.

Characteristic		Overall study population (N=164,221)	Non-PHU^a^ population (n=155,862)	PHU population (n=8359)
**Age group (years), n (%)**				
	0-17	100,811 (61.4)	99,352 (63.7)	1459 (17.5)
	18-64	62,396 (38.0)	55,666 (35.7)	6730 (80.5)
	65+	1014 (0.6)	844 (0.5)	170 (2.0)
Age (years), mean (SD)		19.79 (17.43)	18.79 (16.82)	38.51 (18.01)
Male, n (%)		72,418 (44.1)	69,683 (44.7)	2735 (32.7)
**Race, n (%)**				
	White	41,219 (25.1)	38,762 (24.9)	2,457 (29.4)
	Black	53,872 (32.8)	50,993 (32.7)	2,879 (34.4)
	Other^b^	149 (0.1)	143 (0.1)	6 (0.1)
	Missing^c^	68,981 (42.0)	65,964 (42.3)	3017 (36.1)
**Inpatient visits, n (%)**				
	0	158,763 (96.7)	151,971 (97.5)	6792 (81.3)
	1-5	5,366 (3.3)	3,866 (2.5)	1500 (17.9)
	6-10	74 (<0.1)	20 (<0.1)	54 (0.6)
	11+	18 (<0.1)	5 (<0.1)	13 (0.2)
**Outpatient visits, n (%)**				
	0	3,690 (2.2)	3,663 (2.4)	27 (0.3)
	1-5	95,372 (58.1)	94,138 (60.4)	1234 (14.8)
	6-10	33,745 (20.5)	32,317 (20.7)	1428 (17.1)
	11+	31,414 (19.1)	25,744 (16.5)	5670 (67.8)

^a^PHU: persistent high users.

^b^“Other”describes members of known race/ethnicity not equal to Asian, Hispanic, White, or Black.

^c^“Missing” describes members with empty values for race.

### Latent Class (Cluster) Analyses

LCA models with 2 to 6 classes were trained using the full population to identify the optimal number of classes. The fit statistics for these models were then calculated and compared for the full population (Table 2). The 4-class models were chosen for both the full population and subpopulations as they optimally balanced good model fit with interpretability of the classes (see Multimedia Appendix 5). The LCA’s 4 classes represented probability patterns of diseases and medications that were deemed to be optimal and interpretable for identifying subgroups of patients within the full sample and in each of the diagnostic subpopulations.

A model with the lowest AIC tends to be more complex if it is not the same as the model with the lowest BIC [23]. Thus, we selected the 4-class LCA model since it fit the data better than the 2- and 3-class models, and the classes were more interpretable than those in the 5- and 6-class models (Table 2). Additionally, AIC and BIC metrics can be compared only across nested models (ie, when the terms in one model are a subset of the terms in the other model). As a result, AIC and BIC measures should not be compared across different study subpopulations (Multimedia Appendix 5).

The LCA models were run with 178 different EDCs and RxMGs on the full population and with the same EDCs/RxMGs on the diagnostic subpopulations, excluding the EDCs used to define the subpopulations. Examining all EDCs/RxMGs in our 4-class LCA models, excluding the EDCs used to define our subpopulation, led us to very similar descriptions of each class. A caveat to this observation is that many EDCs/RxMGs had very low or very high probabilities of being observed in all classes and hence were not useful for distinguishing among classes.

**Figure 2:**
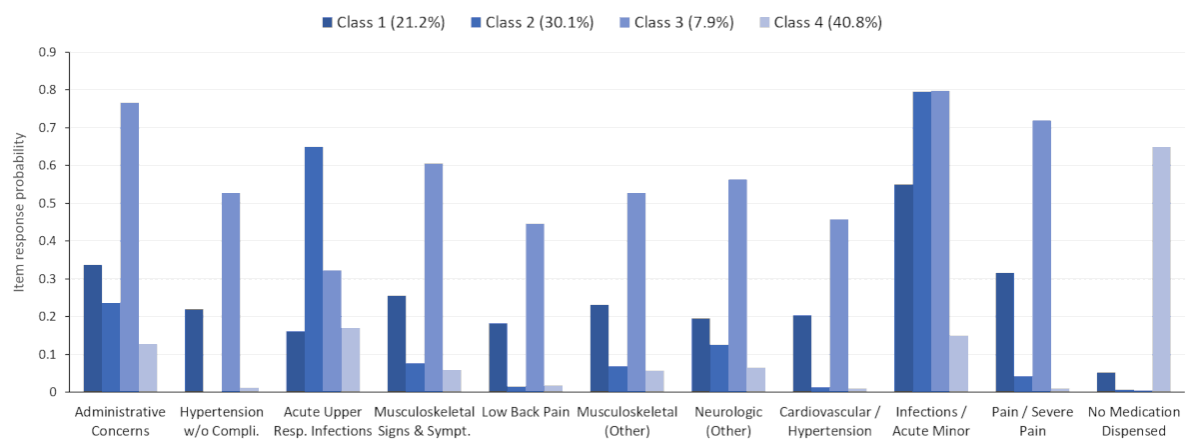
Latent class item-response probabilities for the full population (N=164,221).

Each LCA class contained item-response probabilities for each of the EDC/RxMG codes; however, for only a few of the EDC/RxMG codes, the probability was ≥0.4 in every class. Figure 2 depicts the EDC/RxMG codes that reached the threshold of 0.4 within the full population across all classes. Within the figure, the selected EDC categories that made the threshold are shown along the x-axis and their (item-response) probabilities are shown on the y-axis. The color shading indicates the four different LCA classes, which have different levels of probabilities across different EDCs. Only items with a maximum difference in probability of 0.4 (40%) or greater across pairs of classes are shown for simplicity. Classes 1, 3, and 4 represent people with moderate, high, and low likelihoods of EDCs, respectively. Class 2 is associated with higher probabilities of infections.

**Figure 3:**
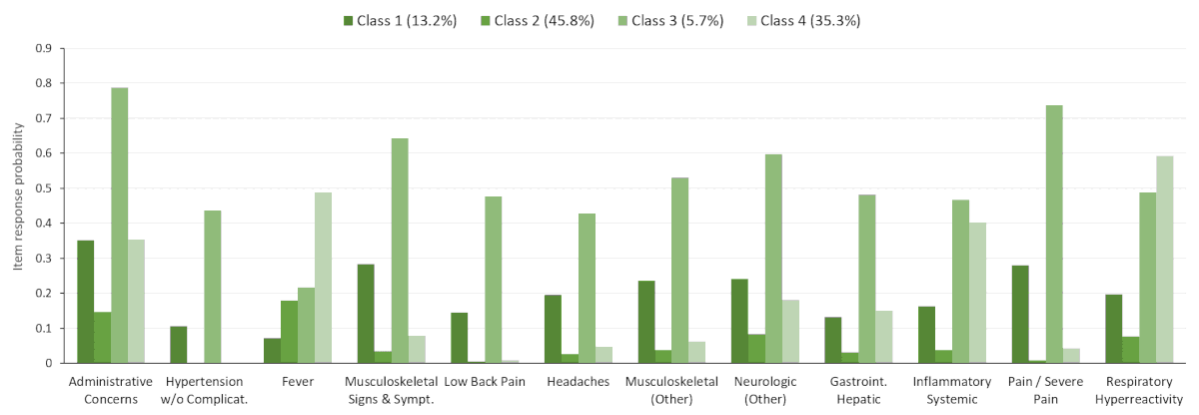
Latent class item-response probabilities for the otitis media subpopulation (n=24,992).

**Figure 4:**
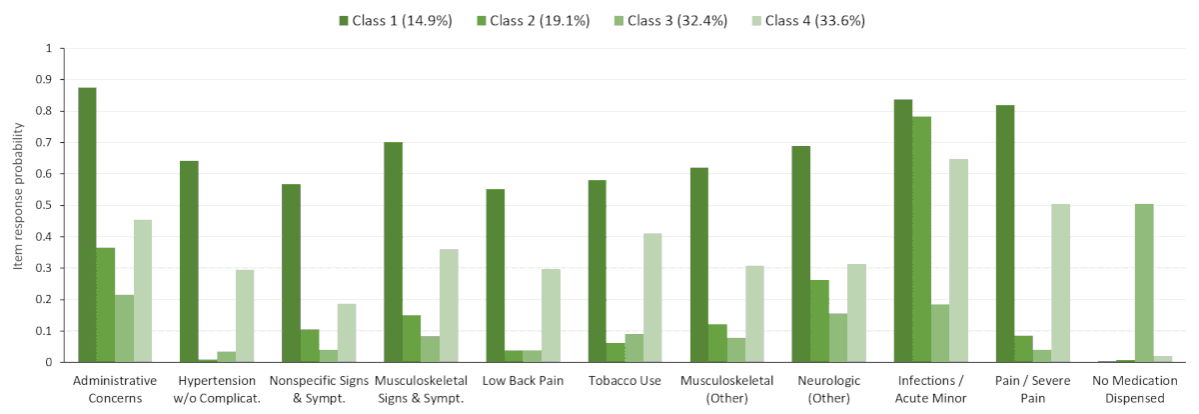
Latent class item-response probabilities for the mental health subpopulation (n=34,456).

**Figure 5:**
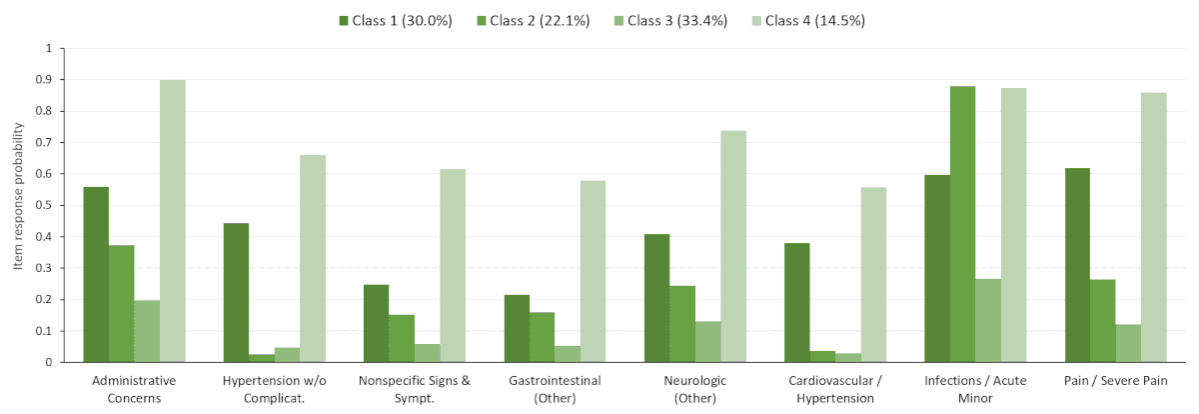
Latent class item-response probabilities for the musculoskeletal subpopulation (n=24,799).

**Figure 6:**
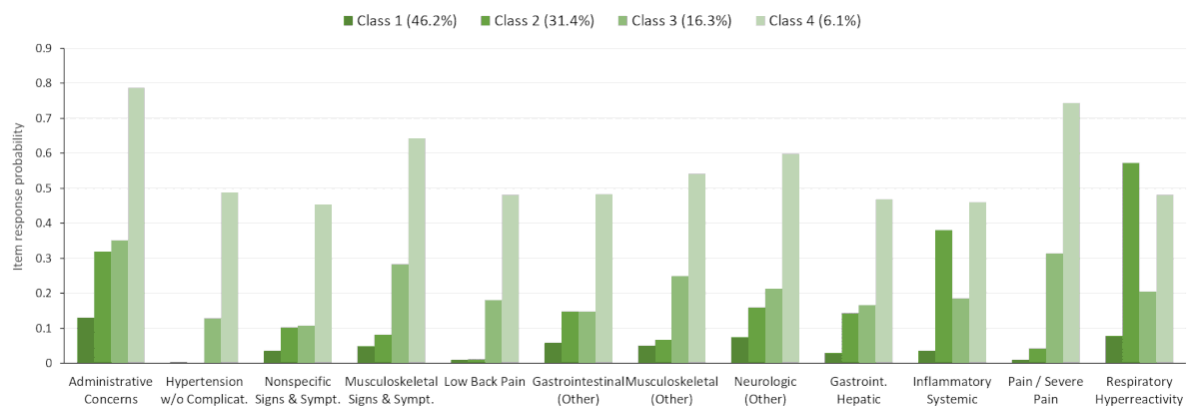
Latent class item-response probabilities for the acute upper respiratory infection subpopulation (n=53,232).

The selected subtype characteristics from the LCA and fractions of patients assigned to each subtype were also explored for each of the four diagnostic subpopulations (Figures 3-6). For example, within the full study population, 21.2% of the patients were attributed to class 1 (Figure 2). However, 13.2%, 14.9%, 30.0%, and 46.2% of the patients were in class 1 for the otitis media (Figure 3), mental health (Figure 4), musculoskeletal (Figure 5), and acute URI (Figure 6) subpopulations, respectively. In Figures 3 to 6, only items with a maximum difference in probability of 0.4 (40%) or greater across pairs of classes are shown for simplicity. In Figure 3, classes 1, 2, and 3 represent people with moderate, low, and high (particularly chronic conditions) likelihoods of EDCs, respectively, whereas class 4 is associated with higher probabilities of infections (eg, URI) and fever. In Figure 4, classes 1 and 3 represent people with high and low likelihoods of EDCs, respectively, whereas class 2 is associated primarily with a high likelihood of minor infections and class 4 represents people with moderate likelihoods of infections and pain. In Figure 5, classes 1, 3, and 4 represent people with moderate, low, and high likelihoods of EDCs, respectively, whereas class 2 is associated primarily with a high likelihood of minor infections. In Figure 6, classes 1, 3, and 4 represent people with low, moderate, and high likelihoods of EDCs, respectively, whereas class 2 is associated primarily with a high likelihood of airway hyperactivity.

Only a handful of EDCs clearly distinguished the four classes in each LCA model (full population and the diagnostic subpopulations). In the full population and in most of the diagnostic subpopulations, three of these classes were associated with uniformly high, moderate, or low probabilities of the EDCs. The remaining class was characterized primarily by a high likelihood of minor infections, pain, or respiratory diagnoses (Figures 2-6).

**Table 2:** Model fit statistics for latent class analysis models with 2 to 6 classes (N=164,221).

Model	G^2^^a^	AIC^b^	BIC^c^
2-class model	5,487,702	9,113,315	9,116,888
3-class model	5,213,964	8,839,935	8,845,300
4-class model	5,088,223	8,714,552	8,721,708
5-class model	4,934,192	8,560,878	8,569,826
6-class model	4,874,634	8,501,679	8,512,419

^a^G^2^: likelihood ratio/deviance statistic.

^b^AIC: Akaike information criterion.

^c^BIC: Bayesian information criterion.

### PHU Predictive Modeling (Logistic Regression)

Logistic regression models were developed for the full population and for each subpopulation to predict PHUs from latent class membership probabilities along with demographic and health utilization characteristics of each patient. These models were trained on a randomly selected sample of 80% of the patients in the full population/subpopulation and were evaluated on a test data set with the other 20% of patients. Classification metrics for each of these models (Table 3) revealed that PHU predictions are more accurate within subpopulations that have a high prevalence of PHUs. For example, the F1-score reached 38.6 in the LCA-enabled regression models predicting PHUs in the full population, whereas the F1-score reached 45.8, 48.7, 48.1, and 43.0 among the otitis media, mental health, musculoskeletal, and acute URI subpopulations, respectively. Although the musculoskeletal subpopulation had the highest percentage of PHUs (Table 3), the regression model for the mental health subpopulation performed the best in terms of the sensitivity and F1-score (62.4 and 48.7 vs 55.1 and 48.1, respectively).

The LCA-enabled regression model for the full population performed modestly lower than the ACG model (ie, F1-score 38.6 vs 48.2); however, the LCA-enabled model had fewer predictors (16 variables) than the ACG model (≥300 variables). The F1-scores of the LCA-enabled regression models in the subpopulations were comparable to the F1-score of the complex ACG model in predicting PHUs in the full population (ie, F1-scores ranging from 43.0 to 48.7 vs 48.2). Since the specificity, sensitivity, PPV, and F1-score were calculated for specific thresholds, only one estimate was calculated for each of those metrics (ie, the 95% CI was not applicable).

**Table 3:** Comparing classification metrics for predicting persistent high user/utilizer (PHU) status.

Metric	Full study population (N=164,221)			Otitis media (n=24,992)		Mental health (n=34,456)		MSK^a^ (n=24,799)	acute URI^b^ (n=53,232)
	ACG^c^	LCA-LRM^d^	LCA-LRM		LCA-LRM		LCA-LRM		LCA-LRM
PPV^e^ (%)	48.60	38.53	44.40		39.91		42.74		41.28
Sensitivity (%)	47.90	38.72	47.23		62.43		55.14		44.99
F1-score (%)	48.20	38.62	45.77		48.69		48.15		43.05
Percentile (threshold)	95th (0.33)	95th (0.33)	95th (0.18)		80th (0.25)		95th (0.53)		95th (0.23)
PHUs (%)	5.1	5.1	4.5		12.8		15.5		4.6

^a^MSK: musculoskeletal.

^b^URI: upper respiratory infection.

^c^ACG: Adjusted Clinical Groups; latent class analysis results not included in the model.

^d^LCA-LRM: latent class analysis-logistic regression model; latent class probabilities included as predictors in the model.

^e^PPV: positive predictive value.

Odds ratios (ORs) of the LCA-enabled regression models predicting PHUs in the full population and in each of the diagnostic subpopulations were calculated separately (Multimedia Appendices 6-10). In all LCA-enabled regression models, the class probabilities were statistically significant in predicting PHUs and resulted in the highest ORs of 22.3, 6.0, and 135.3 for classes 1, 2, and 3 in the full population model, respectively. Other predictors were either not statistically significant (eg, sex, inpatient hospitalization days) or, if significant, had a small effect size (ie, ORs ranging between 0.4 and 3.0). Being Asian or Hispanic, having medical or pharmacy insurance coverage, and being on Medicaid were protective against PHUs (ie, ORs of 0.77, 0.41, 0.85, and 0.69, respectively), while being Black, having a high count of inpatient stays, holding frailty conditions, and likely or possibly experiencing care coordination issues were associated with PHUs (ie, ORs of 1.18, 1.25, 1.14, 1.68, and 3.07, respectively). These findings highlight some of the demographic and health care factors associated with a higher or lower likelihood of being a PHU.

## Discussion

### Principal Findings

PHUs are defined as the patient population who stay in the highest deciles of health care costs and/or utilization for multiple years [1,8-15]. Predicting PHUs is a challenge as their underlying mix of comorbidities and medications may differ across settings [12,13]. To address this analytic gap and improve the efficiency of grouping underlying conditions of PHUs, we applied LCA, a novel unsupervised clustering approach, to the JHHC’s insurance claims data to identify classes of high-utilizing patients with similar probabilities for different sets of diseases and medications. We then explored the value of the LCA classes for predicting which patients, within the full population or specific subpopulations, will become PHUs using a simple parsimonious regression model, and then compared its predictions to those of a more detailed complex predictive model.

Our study demonstrated the use of nontraditional statistical clustering methods such as LCA to facilitate the automated development of diagnostic and medication probability classes that can be effectively used in traditional logistic regression models to predict PHUs, without the need for complex predictive models. Two of our study findings specifically support the use of LCA in predicting PHUs. First, the F1-score of the LCA-enabled logistic regression was comparable to that of the complex predictive model despite having a fraction of the variable predictors (16 vs ≥300 variables). Second, the ORs of the LCA-derived classes were much higher (ranging from 22 to 135) than those of the other variables (ranging from 0.4 to 3.0) used in the logistic regressions. Therefore, LCA can be an efficient (ie, unsupervised process requires minimal manual effort), effective (ie, high ORs in the predictive models), and usable (ie, avoiding complex predictive models) method for predicting PHUs in different settings.

The mix of LCA classes may differ among PHUs of different health systems. For example, our study population of 164,221 patients included 130,711 members enrolled in a special Medicaid insurance plan (ie, Johns Hopkins Priority Partners) targeting mothers and children. Thus, as 79.6% of the study population were enrolled in this Medicaid program, the average age of the full population was close to 20 years. Consequently, the most common EDCs for three of the four diagnostic subpopulations included pediatric conditions such as ear problems [26], which led our clinical experts to categorize one of the subpopulations as otitis media. In addition, the fact that one of the diagnostic subpopulations was identified as “mental health” reflects the reported association of higher health care costs for children with mental health conditions [27], which made this subpopulation particularly relevant to our study of PHUs.

### Comparison With Prior Work

A few prior studies have explored the use of LCA and other classifying techniques to improve the prediction of PHUs. One study focused on US older and middle-aged patients and grouped them using the Medical Expenditure Panel Survey data set to explore high to moderate utilization rates [16]. Due to the older demographic of their population, the study found age, unemployment, insurance status, and number of chronic conditions and medications as key clustering factors. Two separate studies in Singapore applied LCA to segment populations into different utilization classes [18,19]. Their first study focused on primary health care patients enrolled in governmental insurance programs, and found that a specific class with metabolic diseases and multiorgan complications had the highest hospital admissions and ED visits [18]. Their second study focused on patients enrolled in the government-sponsored hospital-to-home transitional care program, and found that patients with frailty and cognitive impairment had the highest hospital readmission rate [19]. Another study in the United States further explored the use of LCA grouping for improving the prediction of superutilizers; however, that study was limited to veterans experiencing homelessness [15]. Veterans who were in an LCA group representing older, male, White, unmarried, and disabled patients proved most likely to be superutilizers. However, none of these studies explored the Medicaid population (with a high percentage of pediatric patients), assessed the LCA classes in separate diagnostic subpopulations in addition to the full population, or compared the value of LCA classes in predicting PHUs compared to a standard/complex utilization prediction model.

### Practical Implications

Health care providers increasingly use risk stratification tools to manage their patient populations. However, providers often do not have access to insurance claims data and use local EHRs to risk stratify patients and predict PHUs [6,7,28]. Despite the advances in using unique EHR data in improving risk prediction [29-34], quality issues render EHR data challenging to use in complex predictive models of utilization [35-38]. Using an unsupervised methodology to classify underlying diagnostic and medications can enable providers to surmount some of these deficiencies and improve the prediction of PHUs using EHR data [37]. Furthermore, LCA and similar classification approaches can help providers to better understand the unique needs of their underlying patient populations and to better target their population health interventions [39]. Nonetheless, fully automating the LCA classes, and excluding clinical feedback in the process, may result in identifying subpopulations that may not provide a meaningful clinical context for targeted care management.

### Limitations

Our study has several limitations. First, the results of our LCA approach, and the improvement of the PHU prediction, may not generalize to other populations (eg, older adults, Medicare), settings (eg, inpatient only), or data sources (eg, EHRs). Future research should explore the use of LCA in new populations and settings using alternate data sources. Second, our specific definition for PHU (ie, percentile of cost and time period) may not fit all populations. The risk stratification research community should offer a harmonized definition of PHU so that various research findings on PHUs can be compared effectively to establish generalizable evidence. Third, results of the logistic regression should be interpreted with caution as race and ethnicity are likely to be closely linked to differences in health care coverage and quality rather than being directly related to PHU [40,41]. Fourth, although the LCA approach automates the classification of the populations, clinical feedback is still key to produce useful results. Hence, the LCA process may become more complex to incorporate in clinical settings compared to the traditional regression models such as ACGs [1,21]. Finally, our selection of the diagnostic subpopulations was based on subjective feedback provided by clinical experts. Future research should examine a mix of qualitative and quantitative methods to normalize and expedite this process. Moreover, with even ideal classification of high-cost health care users, effective operational use of these classes in clinical and operational settings remains to be determined.

### Conclusion

A small percentage of patients use most of the health care services continuously over extended periods. We used LCA, an unsupervised clustering approach, to automate the process of extracting classes of comorbidity and medication probabilities for individual patients that can be effectively used in predicting PHUs. The latent classes highlight broad differences in health care utilization patterns among groups of people, while also providing a way to condense critical information into a smaller set of variables to simplify the PHU prediction model and improve its interpretability. From a care management perspective, the LCA and PHU prediction models provide care managers with insights on specific resource utilization variables that are strongly associated with PHU. Future studies should investigate the value of LCA-derived classes for predicting PHUs in other health care settings with potentially different underlying populations.

## Appendix

Multimedia Appendix 1 Descriptive statistics for the otitis media subpopulation (n=24,992).

Multimedia Appendix 2 Descriptive statistics for the mental health subpopulation (n=34,456).

Multimedia Appendix 3 Descriptive statistics for the musculoskeletal subpopulation (n=24,799).

Multimedia Appendix 4 Descriptive statistics for acute upper respiratory tract infection (URI) subpopulation (n=53,232).

Multimedia Appendix 5 Model fit statistics for latent class analysis (LCA) in diagnostic subpopulations.

Multimedia Appendix 6 Odds ratios of predictors in the LCA-enabled logistic regression model predicting persistent health care users/utilizers (PHUs) in the full population (N=164,221).

Multimedia Appendix 7 Logistic regression odds ratios for the otitis media subpopulation.

Multimedia Appendix 8 Logistic regression odds ratios for the mental health subpopulation.

Multimedia Appendix 9 Logistic regression odds ratios for the musculoskeletal subpopulation.

Multimedia Appendix 10 Logistic regression odds ratios for the acute upper respiratory infection subpopulation.

## Abbreviations

ACG: Adjusted Clinical Groups
AIC: Akaike information criterion
BIC: Bayesian information criterion
CONSORT: Consolidated Standards of Reporting Trials
ED: emergency department
EDC: expanded diagnostic cluster
EHR: electronic health record
JHHC: Johns Hopkins Health Care
LCA: latent class analysis
OR: odds ratio
PHU: persistent high user/utilizer
PPV: positive predictive value
RxMG: prescription-defined morbidity groups
URI: upper respiratory infection
