# Supplementary material for: Assessing the Value of Unsupervised Clustering in Predicting Persistent High Health Care Utilizers: Retrospective Analysis of Insurance Claims Data
Source: JMIR Med Inform. 2021 Nov 25;9(11):e31442. doi: 10.2196/31442 (PMC8663459; doi:10.2196/31442)
Supplement: Multimedia Appendix 1 [file medinform_v9i11e31442_app1.doc]

**Table A1. Descriptive statistics for otitis media subpopulation (N=24,992)**

|  |  | **Overall Population** | **Non-PHU Population** | **PHU  Population** |
| --- | --- | --- | --- | --- |
| **Number** |  | 24,992 | 23,875 | 1,117 |
| **Age** | 0-17 | 21,385 | 20,903 | 482 |
|  | 18-64 | 3,570 | 2,944 | 626 |
|  | 65+ | 37 | 28 | 9 |
|  | Mean | 9.79 | 9.04 | 25.71 |
|  | SD | 12.53 | 11.55 | 19.93 |
| **Sex** | (# Male) | 11,903 | 11,510 | 393 |
| **Race** | White | 8,959 | 8,513 | 446 |
|  | Black | 6,823 | 6,451 | 372 |
|  | Other ^1^ | 30 | 27 | 3 |
| **Inpatient  Visits** | 0 | 24,180 | 23,252 | 928 |
|  | 1-5 | 801 | 619 | 182 |
|  | 6-10 | 11 | 4 | 7 |
|  | 11+ | 0 | 0 | 0 |
| **Outpatient  Visits** | 0 | 409 | 407 | 2 |
|  | 1-5 | 9,695 | 9,620 | 75 |
|  | 6-10 | 7,939 | 7,742 | 197 |
|  | 11+ | 6,949 | 6,106 | 843 |

*^1^ Other Race describes people of known race/ethnicity not equal to Asian, Hispanic, White, or Black.*
