# Supplementary material for: Assessing the Value of Unsupervised Clustering in Predicting Persistent High Health Care Utilizers: Retrospective Analysis of Insurance Claims Data
Source: JMIR Med Inform. 2021 Nov 25;9(11):e31442. doi: 10.2196/31442 (PMC8663459; doi:10.2196/31442)
Supplement: Multimedia Appendix 2 [file medinform_v9i11e31442_app2.doc]

**Table A2. Descriptive statistics for mental health subpopulation (N=34,456)**

|  |  | **Overall Population** | **Non-PHU Population** | **PHU  Population** |
| --- | --- | --- | --- | --- |
| **Number** |  | 34,456 | 30,033 | 4,423 |
| **Age** | 0-17 | 12,707 | 12,306 | 401 |
|  | 18-64 | 21,582 | 17,603 | 3,979 |
|  | 65+ | 167 | 124 | 43 |
|  | Mean | 28.82 | 26.99 | 41.25 |
|  | SD | 17.46 | 17.05 | 14.95 |
| **Sex** | (# Male) | 14,732 | 13,456 | 1,276 |
| **Race** | White | 11,938 | 10,346 | 1,592 |
|  | Black | 11,310 | 9,765 | 1,545 |
|  | Other ^1^ | 23 | 20 | 3 |
| **Inpatient  Visits** | 0 | 31,376 | 28,092 | 3,284 |
|  | 1-5 | 3,006 | 1,922 | 1,084 |
|  | 6-10 | 57 | 14 | 43 |
|  | 11+ | 17 | 5 | 12 |
| **Outpatient  Visits** | 0 | 121 | 118 | 3 |
|  | 1-5 | 12,049 | 11,701 | 348 |
|  | 6-10 | 7,851 | 7,310 | 541 |
|  | 11+ | 14,435 | 10,904 | 3,531 |

*^1^ Other Race describes people of known race/ethnicity not equal to Asian, Hispanic, White, or Black.*
