# Supplementary material for: Assessing the Value of Unsupervised Clustering in Predicting Persistent High Health Care Utilizers: Retrospective Analysis of Insurance Claims Data
Source: JMIR Med Inform. 2021 Nov 25;9(11):e31442. doi: 10.2196/31442 (PMC8663459; doi:10.2196/31442)
Supplement: Multimedia Appendix 3 [file medinform_v9i11e31442_app3.doc]

**Table A3. Descriptive statistics for musculoskeletal subpopulation (N=24,799)**

|  |  | **Overall Population** | **Non-PHU Population** | **PHU  Population** |
| --- | --- | --- | --- | --- |
| **Number** |  | 24,799 | 20,929 | 3,870 |
| **Age** | 0-17 | 7,542 | 7,272 | 270 |
|  | 18-64 | 16,912 | 13,389 | 3,523 |
|  | 65+ | 345 | 268 | 77 |
|  | Mean | 32.60 | 30.61 | 43.35 |
|  | SD | 18.11 | 18.01 | 14.51 |
| **Sex** | (# Male) | 8,940 | 7,929 | 1,011 |
| **Race** | White | 7,421 | 6,104 | 1,317 |
|  | Black | 7,543 | 6,153 | 1,390 |
|  | Other ^1^ | 22 | 21 | 1 |
| **Inpatient  Visits** | 0 | 22,499 | 19,579 | 2,920 |
|  | 1-5 | 2,227 | 1,333 | 894 |
|  | 6-10 | 56 | 12 | 44 |
|  | 11+ | 17 | 5 | 12 |
| **Outpatient  Visits** | 0 | 190 | 189 | 1 |
|  | 1-5 | 6,247 | 6,056 | 191 |
|  | 6-10 | 5,740 | 5,351 | 389 |
|  | 11+ | 12,622 | 9,333 | 3,289 |

*^1^ Other Race describes people of known race/ethnicity not equal to Asian, Hispanic, White, or Black.*
