# Supplementary material for: Assessing the Value of Unsupervised Clustering in Predicting Persistent High Health Care Utilizers: Retrospective Analysis of Insurance Claims Data
Source: JMIR Med Inform. 2021 Nov 25;9(11):e31442. doi: 10.2196/31442 (PMC8663459; doi:10.2196/31442)
Supplement: Multimedia Appendix 4 [file medinform_v9i11e31442_app4.doc]

**Table A4. Descriptive statistics for acute URI subpopulation (N=53,232)**

|  |  | **Overall Population** | **Non-PHU Population** | **PHU  Population** |
| --- | --- | --- | --- | --- |
| **Number** | 53,232 | 50,761 | 2,471 | 53,232 |
| **Age** | 42,157 | 41,307 | 850 | 42,157 |
|  | 10,974 | 9,384 | 1,590 | 10,974 |
|  | 101 | 70 | 31 | 101 |
|  | 12.82 | 11.99 | 29.85 | 12.82 |
|  | 13.69 | 12.79 | 19.34 | 13.69 |
| **Sex** | 23,713 | 22,967 | 746 | 23,713 |
| **Race** | 17,615 | 16,749 | 866 | 17,615 |
|  | 15,800 | 14,902 | 898 | 15,800 |
|  | 62 | 61 | 1 | 62 |
| **Inpatient  Visits** | 51,409 | 49,436 | 1,973 | 51,409 |
|  | 1,785 | 1,313 | 472 | 1,785 |
|  | 33 | 11 | 22 | 33 |
|  | 5 | 1 | 4 | 5 |
| **Outpatient  Visits** | 1,155 | 1,150 | 5 | 1,155 |
|  | 24,355 | 24,150 | 205 | 24,355 |
|  | 15,183 | 14,772 | 411 | 15,183 |
|  | 12,539 | 10,689 | 1,850 | 12,539 |

*^1^ Other Race describes people of known race/ethnicity not equal to Asian, Hispanic, White, or Black.*
