# Supplementary material for: Assessing the Value of Unsupervised Clustering in Predicting Persistent High Health Care Utilizers: Retrospective Analysis of Insurance Claims Data
Source: JMIR Med Inform. 2021 Nov 25;9(11):e31442. doi: 10.2196/31442 (PMC8663459; doi:10.2196/31442)
Supplement: Multimedia Appendix 5 [file medinform_v9i11e31442_app5.doc]

**Table A5. Model fit statistics for LCA - diagnostic subpopulations**

|  | **G^2^  (likelihood ratio /deviance statistic)** | **AIC** | **BIC** |
| --- | --- | --- | --- |
| **Otitis Media (N=24,992)** | 849871 | 1345591 | 1351369 |
| **Mental Health (N=34,456)** | 1924621 | 2633673 | 2639713 |
| **Musculoskeletal (N=24,799)** | 1432460 | 1930653 | 1936425 |
| **Acute URI (N=53,232)** | 1791336 | 2910480 | 2916795 |

*G^2^: likelihood ratio/deviance statistic.*

*AIC: Akaike information criterion.*

*BIC: Bayesian information criterion.*
