# Supplementary material for: Assessing the Value of Unsupervised Clustering in Predicting Persistent High Health Care Utilizers: Retrospective Analysis of Insurance Claims Data
Source: JMIR Med Inform. 2021 Nov 25;9(11):e31442. doi: 10.2196/31442 (PMC8663459; doi:10.2196/31442)
Supplement: Multimedia Appendix 6 [file medinform_v9i11e31442_app6.doc]

**Figure A1. Odds ratios of predictors in the LCA-enabled logistic regression model predicting PHUs in the full population (N=164,221)**

**
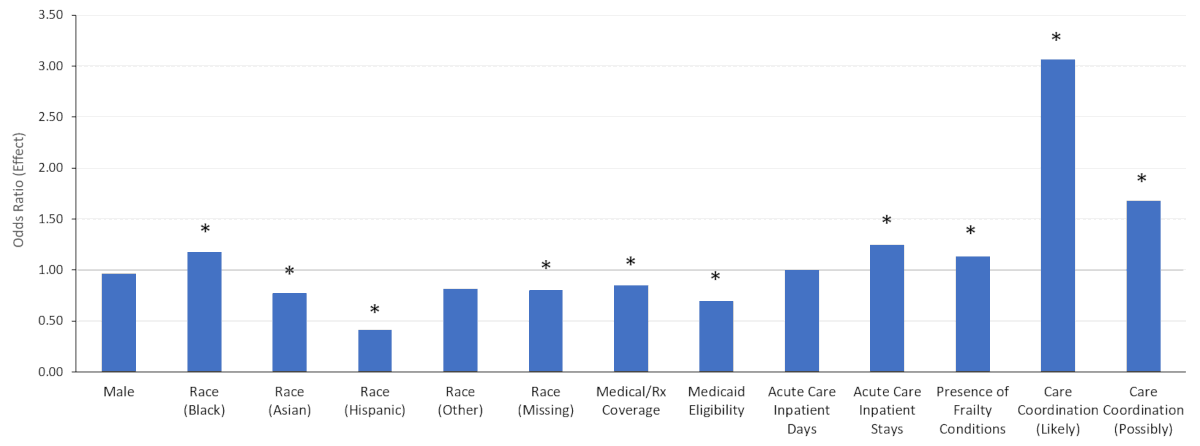
**

*The LCA-class predictor odds ratios (22.3, 6.0 and 135.3) are excluded
to better visualize the magnitude of the remaining predictors.*

*To calculate the ORs, LCA class 4 was used as the reference class.*

*Orange line indicates an odds ratio of 1.*

*The star signs indicate odds ratios with statistical significance.*
