# Supplementary material for: Assessing the Value of Unsupervised Clustering in Predicting Persistent High Health Care Utilizers: Retrospective Analysis of Insurance Claims Data
Source: JMIR Med Inform. 2021 Nov 25;9(11):e31442. doi: 10.2196/31442 (PMC8663459; doi:10.2196/31442)
Supplement: Multimedia Appendix 8 [file medinform_v9i11e31442_app8.doc]

**Figure A3. Logistic regression odds ratios for mental health subpopulation**

**
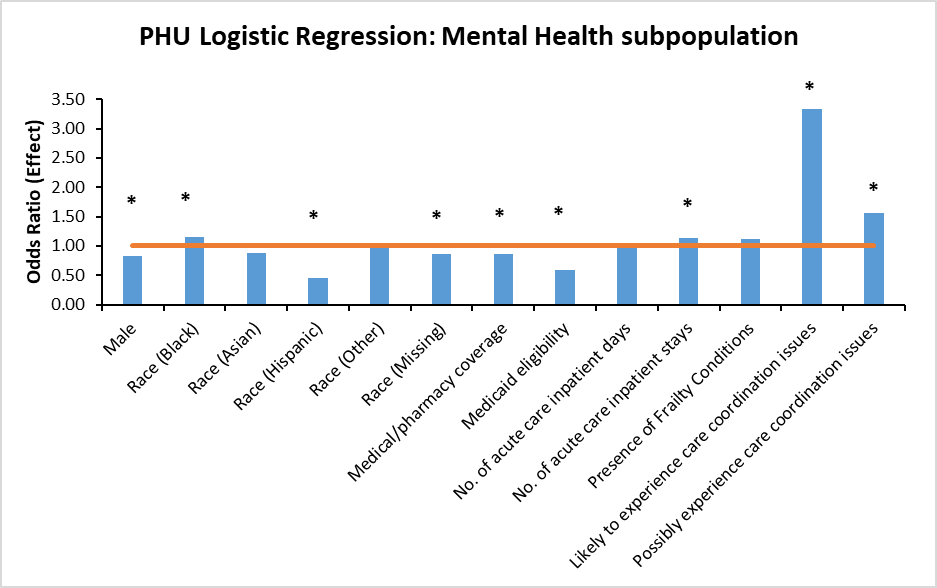
**

*Orange line indicates an odds ratio of 1.*

*The star signs indicate odds ratios with statistical significance.*
